# Understanding Patients’ Preferences for Discussing Sexuality After Surgery—A Qualitative Study of Sexuality and Body Image in Women with Ovarian Cancer

**DOI:** 10.3390/curroncol33020110

**Published:** 2026-02-12

**Authors:** Julia Rosa Stöckl, Marlene M. Lee, Jalid Sehouli, Adak Pirmorady-Sehouli

**Affiliations:** 1 Department of Gynecology with Center for Oncological Surgery, Charité—Universitätsmedizin Berlin, Corporate Member of Freie Universität Berlin and Humboldt-Universität zu Berlin, Augustenburger Platz 1, 13353 Berlin, Germany; marlene.lee@charite.de (M.M.L.); jalid.sehouli@charite.de (J.S.); 2 Department of Psychosomatic Medicine, Charité—Universitätsmedizin Berlin, Corporate Member of Freie Universität Berlin and Humboldt-Universität zu Berlin, Hindenburgdamm 30, 12203 Berlin, Germany; adak.pirmorady@charite.de

**Keywords:** ovarian cancer, sexuality, gynecologic oncology, perioperative care, quality of life

## Abstract

Background: Aspects of sexuality are often deprioritized or overlooked during the treatment of women with ovarian cancer, despite the profound physical and psychosocial impact of surgical and oncological therapies. This study aimed to explore experiences of sexuality and body image in women with ovarian cancer during the peri- and postoperative period. Methods: This qualitative exploratory study was based on twelve semi-structured, in-depth interviews with women aged ≥18 years diagnosed with ovarian cancer. Interviews were conducted in person or by telephone, transcribed verbatim, and analyzed using qualitative content analysis according to Kuckartz with MAXQDA 24.11. Results: Participants described substantial changes in sexuality and body image throughout their disease trajectory. Participants emphasized a lack of attention to sexual health and well-being in ovarian cancer care and expressed a need for timely and sensitive dialogue with medical professionals, social support systems, and with themselves regarding sexuality and intimacy. Conclusions: Sexuality emerged as a key factor for overall well-being and empowerment in women with ovarian cancer, regardless of age or relationship status. The findings suggest that sexuality-related concerns are oftentimes overlooked in clinical care and may negatively influence recovery. Greater awareness and a holistic, patient-centered perspective may help support sexual health and well-being throughout the disease trajectory.

## Simple Summary

Sexuality is still insufficiently addressed in medical settings, in science, and in clinical practice. Ovarian cancer patients seem to be disadvantaged compared to other patient groups, as multivisceral surgical and oncological interventions directly and indirectly affect sexual organs and can have a profound impact on sexual health and quality of life. In this qualitative study, we explored the experiences of twelve women with ovarian cancer to understand how operative as well as conservative interventions (chemotherapy, targeted therapies, cancer care) can affect sexuality and body image, and what role medical professionals may play in supporting patients during this process. We anchor our findings in the importance of timely dialogue with healthcare professionals, the inclusion of partners and/or social support systems, and opportunities for exchange, in order to raise awareness. Regardless of age or life stage, all interview partners described various aspects of their sexuality as important components of recovery and of redefining intimacy in their lives.

## 1. Introduction

Sexuality and body image can be profoundly affected throughout the patient journey, yet they remain insufficiently addressed in clinical practice. While physical side effects of cancer treatment are well documented, research on sexuality and body image, particularly from a qualitative, patient-centered perspective, remains limited. Few studies provide space for patients to explore and articulate what it means to live in a changed body following an ovarian cancer diagnosis and treatment.

Evidence from a study conducted at the Ovarian Cancer Center of Charité Campus Virchow Klinikum suggests that sexuality is associated with quality of life and may be linked to the overall survival of women with ovarian cancer [1].

Accordingly, current European Society of Gynecological Oncology (ESGO) guidelines and several national guidelines recommend “actively exploring the topic sexuality” in clinical care [2,3]. Although recent studies indicate increasing awareness of the importance of sexual health and the responsibility it entails, this knowledge has not been adequately translated into routine clinical practice [4,5]. Barriers are diverse and include taboo, shame, lack of training, and time constraints [6]. Despite its recognized relevance, real-world, patient-centered data on sexuality and body image in women with ovarian cancer, especially qualitative data capturing lived experiences, remain scarce. This study aims to explore experiences of sexuality and body image in women with ovarian cancer during the peri- and postoperative period using a qualitative approach. Through this, we aim to better understand the value of the somatic, psychological, and social dimensions of sexuality from the patients’ perspective. For the purposes of this study, we use the term sexual well-being to refer to patients’ experiences of and satisfaction with their sexual lives, whereas sexual health is applied in its broader definition, encompassing physical, mental, and emotional aspects of health. The term sexuality is used to address the overarching concept of sexual expression, gender identity, and intimacy. Body image is conceptualized as a multifaceted construct that includes perceptual, affective, cognitive, and behavioral dimensions [7,8,9].

## 2. Methods

### 2.1. Study Design, Setting, and Recruitment

This qualitative study used on semi-structured interviews to investigate research questions and to explore patient-centered perspectives relevant to clinical practice that may inform future clinical recommendations.

Participants were recruited from a cohort of 664 ovarian cancer patients who had previously participated in the “KORE—Innovation” trial at the Department of Gynecology, Charité Campus Virchow, Berlin, between July 2020 and September 2024 [10]. The KORE study investigated a multimodal perioperative care pathway, including a personalized prehabilitation approach and the Enhanced Recovery After Surgery (ERAS) pathway, in ovarian cancer patients, and provided the framework for participant identification and recruitment.

As part of the KORE trial, all patients had previously answered four sexuality-related items in the European Organisation for Research and Treatment of Cancer (EORTC) QLQ-OV28 questionnaire at four different time points during the treatment trajectory. For the present study, a subgroup of 65 patients who had completed all sexuality-related items at all four time points was identified and contacted by email. Within 24 h, eight participants agreed to participate. Four additional participants were recruited in a second round using a maximum variation sampling strategy to ensure adequate representation of different age groups within the cohort.

Patients were eligible for the present study if they were aged ≥18 years, diagnosed with ovarian cancer, had undergone surgery and chemotherapy, and were able to participate in an interview conducted in German. Exclusion criteria were age ≤ 18 years, insufficient German language proficiency, severe cognitive impairment, or any condition that could negatively affect participation in an in-depth interview.

### 2.2. Data Collection

In total, twelve interviews lasting 30–45 min were conducted either by telephone or in person at Charité Campus Virchow. Interviews were conducted in German; all participants were proficient in German.

The semi-structured interview guideline was developed following a literature review and based on the biopsychosocial model to capture different dimensions of participants’ experiences, while allowing sufficient space for individual narratives to unfold. The interview guide was developed by a multidisciplinary research team consisting of a gynecologist from the Department of Gynecology at Charité Campus Virchow, a psychosomatic physician from the Department of Psychosomatic Medicine at Charité Campus Benjamin Franklin, and the medical doctoral student who subsequently conducted the interviews. Five interviews were conducted during a pilot phase, after which the research team concluded that the interview guide adequately addressed the research question and was not destabilizing for participants. Based on insights gained during this phase, the guide was further refined with additional questions, and the pilot interviews were included in the final analysis. Example interview questions included Has your sexuality changed during your patient journey? What was your biggest fear regarding your sexuality?

### 2.3. Data Analysis

All interviews were audio-recorded and transcribed verbatim using the integrated transcription tool in MAXQDA 24.11 as well as by the author, who also conducted the interviews. Descriptive statistics of participant characteristics were generated using Pages 12.0.

Data analysis followed the thematic qualitative approach described by Kuckartz [11].

Interviews were analyzed twice in MAXQDA 24.11 using an inductive–deductive coding strategy. Between the analysis phases, a longer break was taken to improve intra-coder reliability. Codes were subsequently organized into code clusters and revised several times until a final category system was defined.

The interviewer was not involved in the clinical treatment of participants. Reflexive protocols were written throughout the research process to document reflections on the interviewing process and potential researcher bias. All analytical steps were supervised by the team of co-authors. Selected interviews were coded together with a supervisor to incorporate additional perspectives. Emerging themes and unexpected findings were discussed within the research team.

Ethical approval for this study was obtained from the Ethics Committee at Charité Campus Benjamin Franklin (EA4/068/25).

## 3. Results

### 3.1. Participant Characteristics

Twelve women with a median age of 55 years, aged between 36 and 83 years, participated in the qualitative interviews. Age at the time of diagnosis had a median of 55 years and ranged from 33 to 81 years.

All participants identified as cisgender women and described themselves as heterosexual. One participant reported uncertainty regarding her gender identity.

At the time of the interview, three participants were single, two were in a relationship, and seven were married. Two participants reported a change in their relationship status during their patient journey.

Four participants were diagnosed with FIGO stage I-II ovarian cancer, and eight with FIGO stage III-IV disease. All twelve participants underwent primary cytoreductive surgery and adjuvant chemotherapy, and nine received maintenance therapy. Four participants experienced disease recurrence. Two women had a stoma.

Detailed patient characteristics are depicted in Table 1.

Among the younger participants, issues related to fertility and motherhood were prominent. One participant was diagnosed during pregnancy, one completed cryopreservation and one was unable to fulfill her wish to have children.

Analysis of the twelve interviews revealed saturation across the identified themes.

### 3.2. Redefining Sexuality and Intimacy

Participants described changes in their sexuality across somatic, psychological, and social dimensions, reflecting a biopsychosocial understanding of sexuality.

While some interview partners reported high psychological distress and a strong need to talk about the challenges they faced, others described a more balanced sexual life and were more focused on contributing their stories in order to help other women. Some participants described changes on a somatic level, while others emphasized psychological aspects that prevented them from fully and freely living their sexual identity. Across all interviews, participants expressed a strong wish to raise awareness.

#### 3.2.1. Somatic Dimension

On a somatic level patients mentioned a need for more information regarding the possible physical changes that could affect their sexuality and body image. Recurring themes including vaginal dryness, weight gain, decreased libido, hair loss, and dyspareunia and vaginal stenosis were described as some of the main challenges following treatment.

*I didn’t know that everything would change, that I would be more or less stitched together…. That really came as a very big surprise to me, that I wasn’t prepared for. (..) I didn’t want to be touched. (..) At the beginning, my husband almost didn’t know where to touch me at all, without me being uncomfortable or with thinking: “Oh, maybe this is going to hurt.” (Table 2: Patientin_5)*

**Table 2:** Sexuality and body image, sub-themes, and illustrative quotes.

Theme	Redefining Sexuality and Intimacy	
Subthemes	Illustrative Quotes	Age at Interview
Somatic Dimension		
Dyspareunia	I didn’t know that everything would change, that I would be more or less stitched together…. That really came as a very big surprise to me, that I wasn’t prepared for. (…) I didn’t want to be touched. (…) At the beginning, my husband almost didn’t know where to touch me at all, without me being uncomfortable or with thinking: “Oh, maybe this is going to hurt.” (Patient_5)	54 years
Psychological Dimension		
Fears	For me it wasn’t clear at all after surgery, what will happen in my relationship. (…) If ever something will be possible again or what? What will be the consequences… that really triggered fears in me. (Patient_6)	55 years
Redefining sexuality	My husband and I always had a lot, a lot and really intense sex and now I feel more like physical love. Yes, exactly. To do it more gently. Like Tenderness. Exactly. I wish for more tenderness. (Patient_1)	54 years
Female identity	I don’t have a problem there, but also I am saying that I am not a real woman anymore. But really I also have a strong feminist background. …and I am not a real woman, I don’t have ovaries, I don’t have a uterus. And I also don’t have female hormones anymore. (Patient_6)	55 years
	For a while it was a bit of a relief…but later…I think this identity as a mother…that was for sure a thing that I had to deal with for a while…that I can’t be a mother anymore. (Patient_12)	36 years
Social Dimension		
Including partners in the process	For him it was also more or less a scary moment. (…) This unknown feeling that it hurts me, was also his fear. With that also the desire of a man of course decreases. You become more insecure, and then you sometimes grit your teeth together. (Patient_5)	54 years
Theme	Body Image	
Subthemes	Illustrative Quotes	Age at Interview
Somatic Dimension		
Reduced sexual desire	You haven’t touched yourself or something like that. And I know, if you imagine, what they have taken out of my body. I always thought, who knows, if that would work again and if I would function again. But after months, maybe half a year later, then I finally had the desire again and then I touched myself. And I realized, that everything works and functions. (Patientin_7)	83 years
Psychological Dimension		
Understanding the new body	Actually you just want to run away from your own body, you think you are in the wrong movie. (Patient_2)	65 years
Understanding the new body	I have sunbathed with my bikini, but not that somebody has seen me, but at the terrace you can’t really see inside, it was the first time. (…) before the body wasn’t (…) with chemo you can’t really go into the sun. Yes, more people have a stoma than you would think. (…) that you can participate a little in life again. Yes, you need something like that. (Patientin_2)	65 years
Social Dimension		
Body awareness	I have never been a person, that hides or that doesn’t undress in front of my husband or something like that. Now I realized that when he comes in during showering, that maybe unconsciously or not, I don’t know, that I avoid situations, where I show myself or like to show it, because I am not fully one with my body yet. (Patientin_5)	54 years
Dating	Since my surgery I didn’t have (…) with a man, I mean I didn’t have contact with a man, who would have, I would say seen me naked. I would say I have problems there. (…) Also I tell them: “I am not the woman anymore I used to be. I have been very sick. And I also talk about it. When they say ‘Man, you have such a nice body!’, then I say, ‘Yes, but it is full of scars and not only the wrinkles in the face.’ (…) I mean, with 83 a woman is also not as smooth as a peach anymore.” (Patient_7)	83 years

#### 3.2.2. Psychological Dimension

Themes on a psychological level varied, but overall, participants described challenges related to adapting to a new phase in life, including redefining sexuality and intimacy with their partners. The loss of a former sense of self was frequently described, followed by the gradual development of a new phase of life or identity. A lack of information about the potential changes in sexuality often triggered fears regarding intimacy and relationships.

*For me it wasn’t clear at all after surgery, what would happen in my relationship. (..) If something would ever be possible again. (..) What the consequences would be. (..) That really triggered fears in me. (Table 2: Patientin_6)*

For some women, the process involved losing their former sexuality and rediscovering intimacy in new ways.

*My husband and I always had a lot, a lot and really intense sex and now I feel more like physical love. Yes, exactly. To do it more gently. Like Tenderness. (Table 2: Patientin_1)*

When asked about their identity as women, participants’ responses were multifaceted and varied on their individual experiences.

*I don’t have a problem there, but also I am saying that I am not a real woman anymore. But really I also have a strong feminist background. … and I am not a real woman. I don’t have ovaries, I don’t have a uterus. And I also don’t have female hormones anymore. (Table 2: Patientin_6)*

For some young participants, the loss of fertility and the need to let go of the possibility of motherhood emerged as particularly significant earlier than anticipated.

*For a while it was a bit of a relief (..) but later (..) I think this identity as a mother (..) that was for sure a thing that I had to deal with for a while (..) that I can’t be a mother anymore. (Table 2: Patientin_12)*

#### 3.2.3. Social Dimension

When investigating the social dimension, a recurring theme was the fear of losing their partners and concerns about how their illness affected their partners’ sexuality and emotional well-being, as well as the need to provide more support to the partners.

*For him it was also more or less a scary moment. (..) This unkown feeling that it hurts me, was also his fear. With that also the desire of a man of course decreases. You become more insecure, and then you sometimes grit your teeth together. (Table 2: Patientin_5)*

### 3.3. Body Image

We also aimed to capture the impact of oncological treatments on patients’ body image. Body image can serve as an important space for expression and representation and is therefore closely linked to a woman’s sense of identity. Treatment-related changes to the body may consequently have a direct and profound influence on patients’ self-esteem.

#### 3.3.1. Somatic Dimension

Some patients described how they reconnected to themselves after surgery and how they had to learn to trust their bodies again.

*You haven’t touched yourself or something like that. And I know, if you imagine, what they have taken out of my body. I always thought, who knows, if that would work again and if I would function again. But after months, maybe half a year later, then I finally had the desire again and then I touched myself. And I realized, that everything works and functions. (Table 2: Patientin_7)*

#### 3.3.2. Psychological Dimension

Participants described the need for patience in learning to understand and adapt to their changed bodies, as well as in coping with the profound impact on their self-esteem. Central to this process was the development of acceptance of the current situation, enabling patients to maintain quality of life.

*Actually you just want to run away from your own body, you think you are in the wrong movie. (Table 2: Patientin_2)*

Several patients described their process of learning how to participate in life again and how the changed body was a challenge in that process.

*I have sunbathed with my bikini, but not that somebody has seen me, but at the terrace you can’t really see inside, it was the first time. (..) before the body wasn’t (..) with chemo you can’t really go into the sun. Yes, more people have a stoma than you would think. (..) that you can participate a little in life again. Yes, you need something like that. (Table 2: Patientin_2)*

#### 3.3.3. Social Dimension

The changes in body image also brought challenges into the relationships of our patients and their self-esteem, which was affected by their procedures.

*I have never been a person, that hides or that doesn’t undress in front of my husband or something like that. Now I realized that when he comes in during showering, that maybe unconsciously or not, I don’t know, that I avoid situations, where I show myself or like to show it, because I am not fully one with my body yet. (Table 2: Patientin_5)*

Participants who were not in a relationship at the time of the interview reported greater vulnerability and fears about not finding a future partner.

*Since my surgery I didn’t have (..) with a man, I mean I didn’t have contact with a man, who would have (..) seen me naked. I would say I have problems there. (..) Also I tell them: “I am not the woman anymore I used to be. I have been very sick. And I also talk about it.” When they say “Man, you have such a nice body!”, then I say, “Yes, but it is full of scars and not only the wrinkles in the face.” (..) I mean, with 83 a woman is also not as smooth as a peach anymore. (Table 2: Patientin_7)*

### 3.4. Perspectives on Support

Further, we investigated how medical professionals and social environments could support patients during recovery.

#### 3.4.1. Patient–Doctor Communication

Participants mentioned the importance of appropriate timing when addressing sexuality during treatment. While the preferred time was determined individually, a general time frame could still be identified. Participants mentioned a period in which they were beginning to return to everyday life. Although some participants indicated that it would have been important to start the dialogue on sexuality earlier, others clearly stated that sexuality was not a relevant topic for them until they started to feel better, later in the course of recovery.

*Those are, I would think, minor anatomical details, that maybe doctors know better, where they could possibly provide assistance to women? “Maybe at the beginning you try this position and approach it little by little.” (Table 3: Patientin_5)*

**Table 3:** Perspectives, sub-themes, and illustrative quotes.

Theme	Perspectives	
Subthemes	Illustrative quotes	Age at Interview
Patient–Doctor Communication	Those are, I would think, minor anatomical details, that maybe doctors know better, where they could possibly provide assistance to women? “Maybe at the beginning you try this position and approach it little by little.” Yes, I would have wished for something like that. (Patient_5)	54 years
Finding support in community	But what I would say is: You don’t need to be afraid that you won’t be a full woman after surgery. Also, even if you can’t have children anymore, you are still a woman (..) This is just from outside. Nobody sees it. Nobody knows it. (Patient_11)	37 years
Internal dialogue	I am happy I can talk to you about it, because it really is a liberation, I would say. Because one talks about it and is also encouraged a bit. I think to myself: “You are doing things right.” (Patient_9)	64 years

When asked about the appropriate timing for conversations with medical professionals, participants’ responses varied. Reported time points ranged from shortly after surgery up to one year after the completion of chemotherapy. Based on participants’ responses, the reported preference clustered around a median of approximately 8–9 months after surgery. Considering an average chemotherapy duration of approximately 6 months, three temporal patterns could be identified: Group 1 (*n* = 2) preferred initial dialogue within 0–1 months after surgery; Group 2 (*n* = 6) between 3 and 8 months after surgery; and Group 3 (*n* = 4) indicated a preference for initiating dialogue between 1 and 1.5 years after surgery.

#### 3.4.2. Finding Support in the Community

When exploring possible tools for educational purposes, the participants mentioned a wide range of options, including brochures, events, podcasts, and apps. Coping strategies described as helpful were physical activity, nutritional approaches, and spiritual practices, as well as the support from friends and family.

Groups for exchanging experiences with other patients were described ambivalently. While such groups were perceived as potentially empowering, they could also be experienced as emotionally challenging, depending on group dynamics and thematic focus. Nevertheless, many participants expressed a wish to share their experiences and offer encouragement to other women who might be struggling. The space for exchange could be a resource for empowerment of the community.

*You don’t need to be afraid that you won’t be a full woman after surgery. Also, even if you can’t have children anymore, you are still a woman (..) This is just from outside. Nobody sees it. Nobody knows it. (Table 3: Patientin_11)*

#### 3.4.3. Internal Dialogue

Lastly, for some participants, the interview itself represented the first opportunity to reflect and verbalize these questions related to their sexuality.

*I am happy I can talk to you about it, because it really is a liberation, I would say. Because one talks about it and is also encouraged a bit. I think to myself: “You are doing things right.” (Table 3: Patientin_9)*

## 4. Discussion

This qualitative study explored experiences of sexuality and body image in women with ovarian cancer. Participants described substantial changes in their sexuality across somatic, psychological, and social dimensions, as well as a strong need for greater awareness, timely communication, and supportive care. Sexuality was described as an important aspect of coping, identity, and recovery, regardless of age or relationship status.

Mitchell et al. recognized sexual well-being as a marker for health equity and therefore suggested implementing this concept into public health strategies, while distinguishing it from the narrow definition of sexual health [8]. This perspective aligns with our findings, which illustrate that sexuality extends beyond physical functioning and plays a broader role in patients’ overall well-being.

A retrospective study by Balint et al., including 644 ovarian cancer patients, demonstrated that women who were less sexually active after treatment felt less attractive and discontented, whereas more sexually active patients showed a more favorable recovery process. Based on these findings, sexuality has been discussed as a relevant factor associated with quality of life as well as overall survival. The study further recommended that sexuality should be addressed routinely in clinical practice and that psycho-oncological support may encourage a better mind–body connection and patient empowerment [1].

In the present study, different dimensions of sexuality were explored through the lens of the biopsychosocial model in order to understand our patients’ lived realities [12]. Although certain topics, such as fertility-related issues, may appear more relevant among younger women, the loss of fertility and the stigma surrounding childless women remained relevant across age groups. Similarly, finding or maintaining intimate relationships was described as an ongoing topic, including for older participants. These findings suggest the importance of further understanding the evolution of sexual health and well-being as a dynamic process throughout the course of life and of addressing unmet needs across different age groups [13,14].

Findings from previous studies are consistent with these observations. In a study by Carmack Taylor and Basen-Engquist et al., including 232 ovarian cancer patients, of whom 47% were undergoing active therapy and approximately half of the patients were sexually active, about 80% reported vaginal dryness, 62% dyspareunia, and 47% little to no sexual desire. The study also included partners, leading to the conclusion that their participation in psycho-oncological care may be beneficial [15].

Similarly, a qualitative analysis conducted at a gynecological clinic in the southeastern United States showed that the majority of interview partners, regardless of age, experienced negative effects on their sexuality following cancer treatment [16]. Distortions of self-image were identified in 45% of patients in a Charité study including 55 patients in an evaluation of sexuality. Self-image perception after cancer treatment was considered an unmet need [17].

Although several studies indicate that healthcare professionals are aware of the importance of sexuality and the responsibility associated with addressing it, this knowledge has not yet been sufficiently implemented in routine clinical practice [4,5]. Barriers to implementation are multifactorial and include taboo, embarrassment, lack of education, and time constraints [6,18].

Several studies and a literature review covering articles from 1980 to 2024 showed similar results, indicating limited awareness of the complex needs for sexual health and well-being, missed opportunities to provide comprehensive survivorship care and a lack of clear knowledge regarding sexual quality of life of ovarian cancer patients [19,20,21,22,23,24,25,26,27].

Recent studies have emphasized the importance of psychosexual education, psychosocial support, and revisions of healthcare policies in addressing these gaps [28]. Other beneficial therapeutic concepts described in the literature include psycho-oncological consultation, cognitive behavioral therapy, hypnosis, and physical activity [6]. Digital interventions, such as the Gyneas support program, have also been developed to enhance women’s sexual well-being, with a particular focus on empowerment rather than sexual function alone [29]. While participants in our study also described the potential benefits of peer support groups, they emphasized that such settings could be both empowering and emotionally challenging, and we therefore suggest that appropriate facilitation, potentially through a psychotherapist, may be necessary.

The findings further highlight the importance of considering not only medical factors, but also the patients’ personal journeys of patients, including the interaction between these two aspects, as well as the potential influence of biographical experiences during that period. Highlighting these interactions may help to better understand the patients’ individual needs of patients and support the development of more personalized therapeutic concepts [30].

Sexuality should be understood as a multidimensional concept. The World Health Organization (WHO) defined sexual health in 2006 as “a state of physical, emotional, mental and social well-being in relation to sexuality; it is not merely the absence of disease, dysfunction or infirmity” [7].

Accordingly, sexuality encompasses communication with partners, quality of relationships, and the perception of one’s own gender identity [12,31]. These dimensions were reflected in our interviews, in which participants described somatic, psychological, and social aspects of sexuality as closely intertwined throughout their disease trajectory. From a broader perspective, this conceptual framework also highlights the importance of sociocultural aspects and the unmet needs of sexual minorities as emphasized in previous studies [6].

Participants emphasized the importance of timing when addressing sexuality in clinical care. Rather than identifying a single optimal moment, their experiences suggest that the period between oncological treatment and follow-up care may be a particularly relevant time to initiate dialogue. Improved communication between departments and tailored, patient-centered approaches may help reduce feelings of being left alone when additional support is needed. Therefore, alternative conversation starters may be helpful. Previous studies have suggested the use of a pre-appointment questionnaire prior to consultation, allowing patients to indicate whether they wish to talk about sexuality [28].

The appropriate timing for addressing sexuality is inherently individual and should be determined collaboratively by the patient and the clinician. A stable and trusting physician–patient relationship is therefore essential, as it facilitates the identification of an appropriate moment to initiate such conversations. Based on our findings, we recommend that once the patient is somatically stabilized and a treatment plan has been established, a conversation about sexuality may be initiated. This can be supported by open-ended questions that allow patients to articulate their experiences in their own words.

Given the intimate nature of the topic and the potential for shame or discomfort, there is a risk that clinicians may hesitate to reintroduce the subject once it has been avoided. It is therefore crucial that healthcare professionals approach these conversations without fear or embarrassment and recognize it as their responsibility to address and revisit the topic proactively. If the moment is not right for the patient, the question should be raised again after several months.

Examples of suitable open-ended questions include “How have you been feeling about your sexuality?” or “Can you describe what your sexuality was like before treatment, and whether you feel it has changed as a result of therapy?”

Based on our data, we suggest the first 3–8 months following surgery as a potential time frame for initiating such discussions, with approximately 12 months postoperatively serving as an alternative or second opportunity. These conversations should also include information about available support options, such as counseling services, specialized advisory centers, and pelvic floor physiotherapy, as well as assistance with referral to these services.

Overall, our data indicate a general need for dialogue around sexuality among all patients. However, younger patients appeared to benefit most from such discussions, a finding that is consistent with previous studies [28]. Other subgroups within our collective were, for example, women with a stoma, who have specific needs regarding the changes to their body image [6], and the impact of relationship status showed specific needs as well. However, these are only suggestions based on our study and research, and we therefore recommend further investigation to explore these possibilities further.

Overall, these findings suggest that qualitative insights into patients’ experiences can complement existing evidence and help inform future clinical approaches. We therefore suggest that these aspects may be considered within the context of general guidelines for the treatment of ovarian cancer patients and that future prospective studies further explore appropriate time frames and approaches.

## 5. Strengths and Limitations

### 5.1. Strengths

The strengths of this study lie in the inclusion of participants from different age groups. The sample also included different FIGO stages, primary and recurrent disease, various surgical morbidity factors and participants with a stoma. This diverse sample allowed for an exploration of experiences across different levels of sexual activity and sexual well-being. This approach increased data depth and clinical relevance by allowing the inclusion of a broad range of perspectives. However, this clinical heterogeneity may also be considered a limitation, as the specific needs of individual subgroups were not fully explored. Differences in factors influencing sexuality and body image may therefore limit the clinical interpretability of the findings and should be examined in future studies focusing on specific subgroups. Such studies could later be integrated to allow more refined, stratified analyses. Notably, despite the heterogeneity of the patient population, all women expressed a desire to talk about these issues and responded openly and with a high degree of intimacy.

### 5.2. Limitations

The sample size (*n* = 12) limits the generalizability of the findings; given existing research in this field, the results may indicate relevant trends and directions.

In our recruiting process, we chose to identify a patient collective that demonstrated openness and reflectiveness regarding the topic by answering four questions about sexuality during the KORE Innovation program. Therefore, we ensured that these interview partners had already reflected on the topic throughout their journey and could report their experiences retrospectively, and that they also showed motivation to participate in an intimate interviewing situation, which is a challenge in itself. However, this procedure also created a filter, as we were mainly attracting women already connected to the topic already, but we could not capture the experiences of patients who were more introverted or reluctant to address the topic. More public and clinical awareness of the topic, however, could help to create more space to include introverted women and open the door to new studies. Moreover, prevailing societal constructions of sexuality, frequently shaped by commercialization and idealized imagery, risk constraining the understanding of sexuality to narrow norms, thereby amplifying shame and inhibiting open dialogue about diverse and non-normative experiences.

Although most participants in this study were of German nationality, some diversity in national and ethnic backgrounds was present. Due to the small sample size, a differentiated analysis of cultural influences on sexuality was limited. Nevertheless, reporting these characteristics remains important given the underrepresentation of diverse populations in oncological research.

Participation was limited to German-speaking individuals, thereby restricting demographic representation and excluding communities with insufficient language proficiency. In addition, the sample did not include transgender individuals or women in same-sex relationships; therefore, perspectives of lesbian, gay, bisexual, transgender, queer, intersex, asexual, and more (LGBTQIA+) patients were not captured. Further research ins warranted to address the specific needs and challenges of these communities within the healthcare system is warranted.

To provide participants with sufficient space for reflection, we deliberately chose a retrospective interview approach, as it allowed patients to develop an overall perspective on the perceived impact of treatment after its completion. In retrospect, however, longitudinal or concurrent assessments during ongoing therapy may be both feasible and valuable in future studies, enabling a more nuanced understanding of temporal changes in sexuality and body image.

*Qualitative interviews are inherently limited in their ability to identify a specific optimal timing for addressing sexuality, particularly as most questions were retrospective. Future research is needed to further explore and validate appropriate approaches and time frames.*

## 6. Conclusions

The aim of this study was to offer new perspectives and to raise awareness of the sexual quality of life during the treatment of women with ovarian cancer. Participants described sexuality and body image as relevant aspects of their lives across different ages and stages of disease. By integrating broader definitions of sexual health and well-being into medical practice, space can be created for patients to be seen and supported in all dimensions of their lived experience.

Sexuality may change over the course of life, and traumatic experiences can have a significant impact on how this aspect of identity is expressed. Nevertheless, individuals remain sexual beings despite disease. Surviving ovarian cancer does not equate to losing sexual identity, and this dimension of the patient journey deserves acknowledgment, alongside other aspects of recovery.

These findings suggest that sexuality, as well as the appropriate timing for addressing it, may represent an important component of recovery, particularly during the phase in which patients begin to re-engage with everyday life and adapt to bodily changes following treatment [1]. Future studies are needed to further evaluate the impact of supportive approaches and to explore how such considerations may be integrated into clinical practice.

## Figures and Tables

**Table 1:** Patient characteristics.

	*N* = 12
Age	
Median (Range)	55 years (36–83 years)
Education	
Secondary School	*n* = 6
High School Diploma	*n* = 2
University	*n* = 4
Diagnosis	
Primary	*n* = 8
First Recurrence	*n* = 4
Treatment	
Surgery	*n* = 12
Chemotherapy	*n* = 12
Maintenance Therapy	*n* = 9
FIGO	
FIGO I–II	*n* = 4
FIGO III–IV	*n* = 8

## Data Availability

The datasets presented in this article are not readily available because of privacy and ethical limitations for qualitative interviews. Requests to access the datasets should be directed to the authors.
